# Supplementary figures and images for: Time of Postharvest Ethylene Treatments Affects Phenols, Anthocyanins, and Volatile Compounds of Cesanese Red Wine Grape
Source: Foods. 2021 Feb 3;10(2):322. doi: 10.3390/foods10020322 (PMC7913538; doi:10.3390/foods10020322)

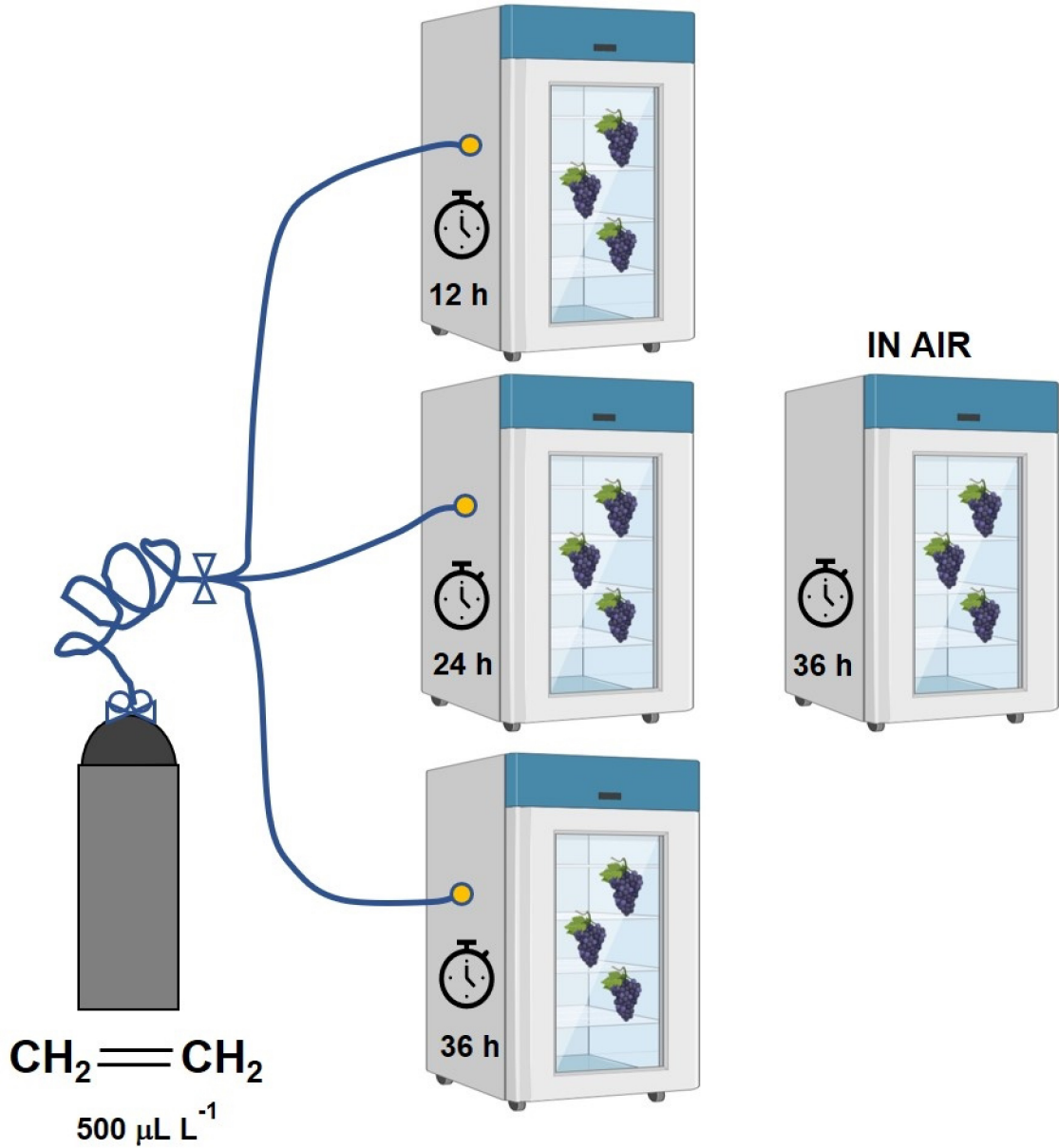

Supplement: Supplementary file 1 [file foods-10-00322-s001.pdf]
